# Specific *in vitro* interferon-gamma and IL-2 production as biomarkers during treatment of chronic Q fever

**DOI:** 10.3389/fmicb.2015.00093

**Published:** 2015-02-12

**Authors:** Teske Schoffelen, Marjolijn C. Wegdam-Blans, Anne Ammerdorffer, Marjolijn J. H. Pronk, Yvonne E. P. Soethoudt, Mihai G. Netea, Jos W. M. van der Meer, Chantal P. Bleeker-Rovers, Marcel van Deuren

**Affiliations:** ^1^Department of Internal Medicine, Radboud University Medical CenterNijmegen, Netherlands; ^2^Department of Medical Microbiology, Laboratory for Pathology and Medical MicrobiologyVeldhoven, Netherlands; ^3^Department of Internal Medicine, Catharina HospitalEindhoven, Netherlands; ^4^Department of Internal Medicine, Elkerliek HospitalHelmond, Netherlands

**Keywords:** Q fever, *Coxiella burnetii*, cell-mediated immunity, interferon-gamma, interleukin-2, biomarker, serology, treatment

## Abstract

**Background:** Antibiotic treatment of chronic Q fever is cumbersome and of long duration. To monitor treatment, there is a need for alternative biomarkers. *Coxiella burnetii*-specific interferon (IFN)-γ and interleukin (IL)-2 production reflect the type of effector and memory T-cell response. In chronic Q fever, *C. burnetii*-specific IFN-γ production is higher and IL-2 production is lower than in individuals with past Q fever. Here we explore whether *C. burnetii*-specific IFN-γ and IL-2 production correlate to treatment response.

**Methods:** We studied the longitudinal *C. burnetii*-specific IFN-γ/IL-2 ratio in fifteen proven chronic Q fever patients. All patients were followed for at least 18 months during antibiotic treatment. Treatment was considered successful when clinical recovery was observed, a positive PCR for *C. burnetii* DNA in blood became persistently negative, anti-phase I IgG showed a fourfold decrease or more, and imaging techniques showed disappearance of infectious foci.

**Results:** Overall, the IFN-γ/IL-2 ratio declined when patients experienced a successful treatment outcome. When treatment failed, IFN-γ/IL-2 ratios did not significantly decrease. The median (±IQR) slope of the longitudinal IFN-γ/IL-2 ratio with successful treatment was -2.10 (-7.02 to -0.06), and -0.15 (-1.13 to 0.25) with unsuccessful treatment (*P* = 0.19). Q fever endocarditis patients had higher IFN-γ/IL-2 ratios than patients with endovascular infections.

**Conclusion:** We propose that the IFN-γ/IL-2 ratio can be used as an additional biomarker for monitoring chronic Q fever treatment, with declining ratios being indicative of successful treatment.

## INTRODUCTION

Q fever is a zoonosis, caused by the Gram-negative, intracellular bacterium *Coxiella burnetii* (Raoult et al., 2005; Parker et al., 2006). Following primary infection, 1–5% of patients develop chronic infection, which can become clinically overt months to years later (Maurin and Raoult, 1999). Endocarditis and infection of a vascular aneurysm or prosthesis are the most common manifestations of chronic Q fever (Botelho-Nevers et al., 2007; Million et al., 2010). Pre-existent cardiac valvular abnormalities, aortic aneurysms, vascular grafts, and immune-compromised state are risk factors (Raoult et al., 2000; Fenollar et al., 2001; Landais et al., 2007).

Clinical symptoms of chronic Q fever are often non-specific, and the diagnosis relies on identifying pre-existing risk-factors, the results of anti-*C. burnetii* serology and PCR for *C. burnetii* DNA on blood or tissue, and results of imaging techniques (Wegdam-Blans et al., 2012a; Anderson et al., 2013). Untreated chronic infection leads to severe morbidity, with a mortality up to 60% (Million et al., 2010). Long-term antibiotics, preferably doxycycline combined with hydroxychloroquine (Raoult et al., 1999; Kersh, 2013), are required to eliminate *C. burnetii*. Antibiotics should be administered for at least 18 months or, in case of a valvular/vascular prosthesis, for at least 24 months (Million et al., 2010). Surgical intervention to replace an infected vascular aneurysm/graft or cardiac valve is often necessary, either in the acute situation of a symptomatic aortic aneurysm or heart failure, or when a patient does not improve on antibiotics (Botelho-Nevers et al., 2007; Kampschreur et al., 2012; Wegdam-Blans et al., 2012b).

The discontinuation of antimicrobial therapy strongly depends on the results of follow-up imaging. In case of vascular infection focus, ^18^F-fluorodeoxyglucose Positron Emission Tomography/Computed Tomography (FDG-PET/CT) is preferred, which has high sensitivity and specificity for low-grade vascular infections (Merhej et al., 2012; Barten et al., 2013). In cases of Q fever endocarditis, vegetations are difficult to detect on echocardiography, and a negative echocardiogram does not rule out endocarditis (Maurin and Raoult, 1999). Therefore, serology is an important tool during the antibiotic treatment of chronic Q fever. Chronic infection is characterized by high titres of anti-phase I IgG antibodies. It is assumed that antibiotic treatment should be continued until these titres have declined at least fourfold, or until titres are below 1:800 in immunofluorescence assay [IFA, or below 1:1024 in a commercial available IFA (Focus Diagnostics)]; (Million et al., 2010). In daily practice, the slow serological decline requires longer treatment, and titres often remain above 1:800 (or above 1:1024 respectively) for a prolonged time.

These limitations show the need for additional biomarkers to monitor treatment of chronic Q fever. In this respect, laboratory tests measuring cell-mediated immune responses may be of value. Interferon-gamma (IFN-γ) plays a pivotal role in the immune response against the intracellular *C. burnetii* (Dellacasagrande et al., 1999; Andoh et al., 2007). Analogous to IFN-γ release assays (IGRAs) that are widely used in *Mycobacterium tuberculosis* infection (Pai et al., 2004), we previously used whole-blood assays to show that the *C. burnetii*-specific IFN-γ production is significantly increased in people that have been exposed to *C. burnetii* (Schoffelen et al., 2013). The interpretation of IFN-γ production in chronic Q fever is complex, since IFN-γ production is a marker of both immunity and infection. We demonstrated, by measuring a broad panel of cytokines, that *ex-vivo C. burnetii*-specific IFN-γ production is higher and interleukin (IL)-2 production is lower in chronic Q fever patients than in patients with past Q fever (Schoffelen et al., 2014), and concluded that a high IFN-γ/IL-2 ratio has a high specificity to discriminate between these two groups.

The present study follows the hypothesis that the IFN-γ/IL-2 ratio will decline during effective treatment of chronic Q fever. To test this, we followed 15 chronic Q fever patients for at least 18 months during antibiotic treatment and performed whole-blood stimulation assays with measurement of IFN-γ and IL-2 on a regularly basis. This study is the first evaluation of a cell-mediated immunity biomarker for treatment of chronic Q fever.

## MATERIALS AND METHODS

### PATIENTS AND FOLLOW-UP

Fifteen chronic Q fever patients, recruited from participating hospitals, were followed in this study for at least 18 months. The study was approved by the Medical Ethical Committee Arnhem-Nijmegen and written informed consent was obtained from all subjects. At the time of diagnosis, all patients had a positive PCR in blood, serum and/or tissue, and anti-phase I IgG titres ≥ 1:1024 (in the absence of acute Q fever). Four patients were diagnosed with endocarditis according to the modified Duke criteria (*n* = 4), and eleven had vascular infection. All fulfilled the criteria of ‘proven chronic Q fever’ of the Dutch consensus group on chronic Q fever (Wegdam-Blans et al., 2012a). Patients were included at different time points after start of treatment. The start of antibiotic treatment was designated *t* = 0. The three patients that were included at the start of treatment or within 2 months after start of treatment are described in more detail. Treatment was considered successful when clinical recovery was observed, a positive PCR for *C. burnetii* DNA on blood became persistently negative, anti-phase I IgG showed a fourfold decrease or more (related to the maximum titre), and imaging techniques showed disappearance of any (vascular or valvular) infectious foci.

### WHOLE BLOOD INCUBATION

Venous blood drawn into 5 mL endotoxin-free lithium-heparin tubes (Vacutainer, BD Biosciences) was processed within 12 h. Blood was aliquoted in separate tubes and incubated at 37^∘^C for 24 h with heat-inactivated *C. burnetii* Nine Mile (NM) RSA493 phase I (Seshadri et al., 2003), mitogen (positive control) or without (negative control).

*C. burnetii* NM was used in an end-concentration of 10^7^ bacteria/mL. Bacteria were cultured in a BSL-3 facility at the Central Veterinary Institute (Lelystad, the Netherlands) as previously described (Schoffelen et al., 2013) and kindly provided by Dr. H. J. Roest. The same batch was used for all assays. The mitogen phytohemagglutinin (PHA, Sigma-Aldrich, St. Louis, MO, USA; 10 μg/mL) was used as a positive control. After incubation, blood cultures were centrifuged at 4656 *g* for 10 min and supernatants were stored at -20^∘^C until assayed.

### CYTOKINE MEASUREMENTS

Interferon-γ concentration in supernatants was measured using a commercial enzyme-linked immunosorbent assay (ELISA; Pelikine compact, Sanquin, Amsterdam, the Netherlands) as previously described (Schoffelen et al., 2014). The background IFN-γ response of the negative control aliquot was subtracted from the stimulated aliquots for each individual sample. In all negative control aliquots, the highest IFN-γ concentration was 46 pg/mL, which was considered acceptable. In addition, all samples showed a net IFN-γ production >24 pg/mL in either the positive control aliquot or in the *C. burnetii-*stimulated aliquot. Thus, all samples were considered valid. IL-2 concentrations in supernatant of the *C. burnetii-*stimulated aliquots were measured using luminex magnetic beads assay (Merck Millipore, Billerica, MA, USA) according to the manufacturer’s instructions.

### ANALYSIS

Graphpad Prism (Graphpad software Inc., version 5) was used to make the graphs and to analyze the data. Non-linear regression to a straight line with least square fit was performed to obtain the best-fit slope of the IFN-γ/IL-2 ratio of each patient over time. Median ± interquartile range (IQR) slope was compared between groups.

## RESULTS

### CASE 1: A PATIENT WITH *C. burnetii*-INFECTED VASCULAR PROSTHESIS WITH SUCCESSFUL TREATMENT

**FIGURE 1:**
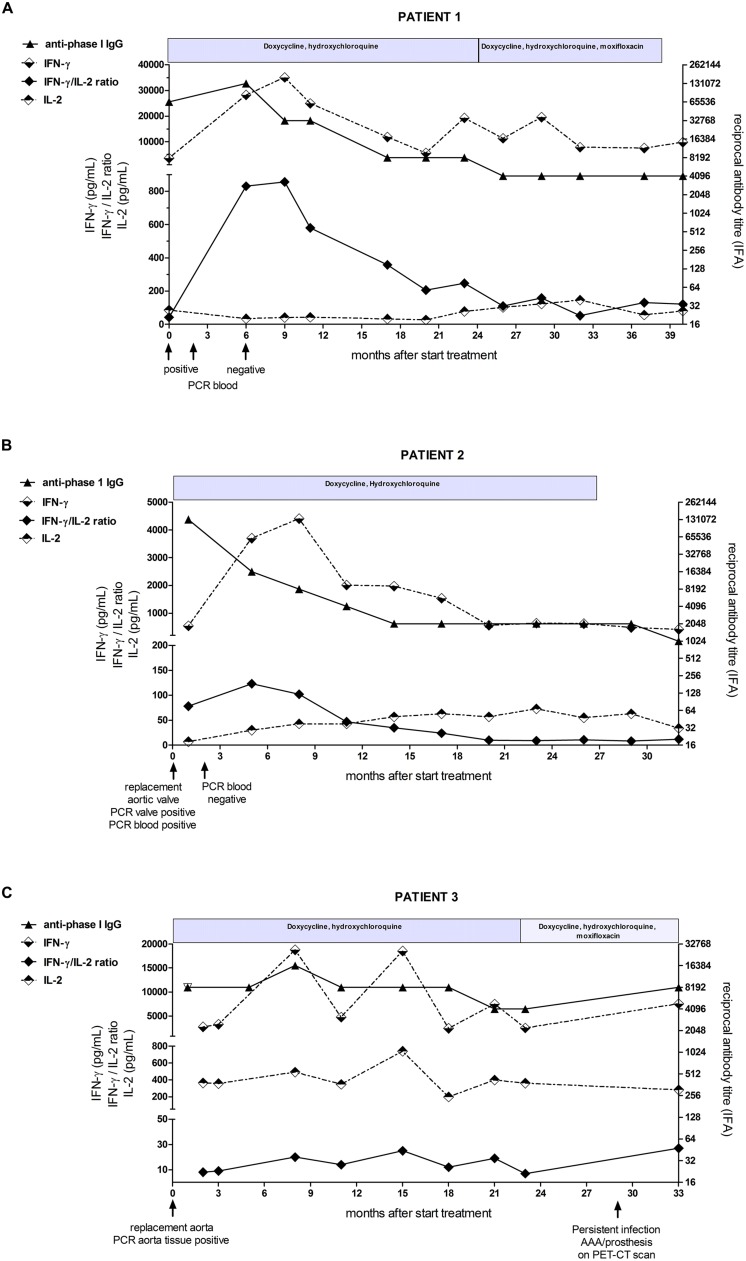
**Detailed overview of immunological parameters in **(A)** patient 1 **(B)** patient 2, and **(C)** patient 3 from start of antibiotic treatment to end of study follow-up.** The IFN-γ and IL-2 concentrations (dotted lines) and the IFN-γ/IL-2 ratio (black diamant) are shown on the left *y*-axis, the anti-phase I IgG antibody-titer (black triangle) is shown on the right *y*-axis. The course of antibiotic treatment is depicted above each graph.

A 30-years-old man with a medical history of traumatic rupture of the thoracic aorta, for which he had undergone vascular surgery receiving an endoprosthesis, suffered from new symptoms of malaise, night sweats, weight loss, and chills. Because of the preceding Q fever epidemic, serology for *C. burnetii* was performed, revealing chronic infection with very high IgG titers against phase I and II (both 1:65536). PCR on serum for *C. burnetii* DNA was positive. He was not aware of a preceding acute Q fever episode. FDG-PET/CT showed no signs of infection at the thoracic aortic prosthesis, nor elsewhere. Transesophageal echocardiogram (TEE) showed mild aortic valve insufficiency without vegetations. Although infection of the vascular prosthesis could not be detected with FDG-PET/CT, it was considered the most likely focus of infection. The patient started antimicrobial therapy with doxycycline and hydroxychloroquine after which he made a quick clinical recovery. PCR in serum became permanently negative 5 months after start of therapy. Although anti-phase I IgG titers had decreased from 1:131072 to 1:8192 in 24 months, titers did not decrease further and moxifloxacin was added to the therapy. This regimen was continued for another 14 months, after which it was decided to stop treatment and continue follow-up 3-monthly. The follow-up –6 months so far– was uneventful. This patient, having a good response on the antimicrobial therapy, showed peaking of the IFN-γ/IL-2 ratio during the first 9 months. Thereafter, the ratio declined and was stable at lower values during the last phase of the treatment, analog to the anti-phase I antibody titers (**Figure 1A**).

### CASE 2: A PATIENT WITH *C. burnetii* ENDOCARDITIS WITH SUCCESSFUL TREATMENT

A 66-years-old man had a history of aortic valve stenosis with valvular replacement (homograft) 11 years before. He presented with symptoms of cardiac failure, for which a TEE was performed, which showed vegetations on the valvular prosthesis. Screening for *C. burnetii* revealed chronic Q fever infection with anti-phase I and II IgG 1:131072 and a positive *C. burnetii* PCR in blood. Therapy with doxycycline and hydroxychloroquine was started and the patient underwent valvular replacement. PCR on valve tissue was positive for *C. burnetii* DNA. After 2 months therapy, PCR on blood was negative and remained so in the follow-up. The patient recovered well. Although he suffered from side effects (mainly photo-sensitivity), the antimicrobial therapy could be continued for 27 months, after which 3-monthly follow-up continued. By that time, the anti-phase I IgG had declined to 1:2048. After stop of antimicrobial therapy, the follow-up has been uneventful (13 months so far).

The IFN-γ/IL-2 ratio showed an initial increase, but from 5 months after start of treatment onward, the IFN-γ/IL-2 ratio declined to very low values (**Figure 1B**).

### CASE 3: A PATIENT WITH *C. burnetii*-INFECTED VASCULAR PROSTHESIS WITH FAILURE OF TREATMENT

In the aftermath of the Q fever epidemic, a 64-years-old man presented with an acute aneurysm of the abdominal aorta. In the preceding weeks, he had back pain, fatigue, malaise, and weight loss. He underwent surgery with placement of a vascular prosthesis, and PCR on aorta tissue was positive for *C. burnetii*. Serology revealed elevated titers of anti-phase I and phase II IgG both 1:8192. PCR for *C. burnetii* DNA in blood was negative. He could not recall a preceding episode of fever or pneumonia. Doxycycline and hydroxychloroquine treatment was started. A transthoracic echocardiogram (TTE) showed thickening of the aortic valve. The patient refused to undergo TEE. He recovered well after the operation. However, anti-*C. burnetii* IgG titers did not decrease in the subsequent 2 years. PCR for *C. burnetii* DNA on blood, performed 3-monthly, remained negative. Moxifloxacin was added to the therapy after 23 months. FDG-PET/CT showed increased uptake at the vascular prosthesis, indicating a persistent infection. This patient showed an unsuccessful treatment of a chronic Q fever vascular infection. The IFN-γ/IL-2 ratio was relatively low from the start of therapy in this patient. The ratio showed an increase after the operation after which it remained mildly elevated, with no tendency to decline (**Figure 1C**). This was the result of neither decrease in IFN-γ production nor increase in IL-2 production over time.

### AN OVERVIEW OF THE IFN-γ/IL-2 RATIO IN FOLLOW-UP OF 15 CHRONIC Q FEVER PATIENTS

**FIGURE 2:**
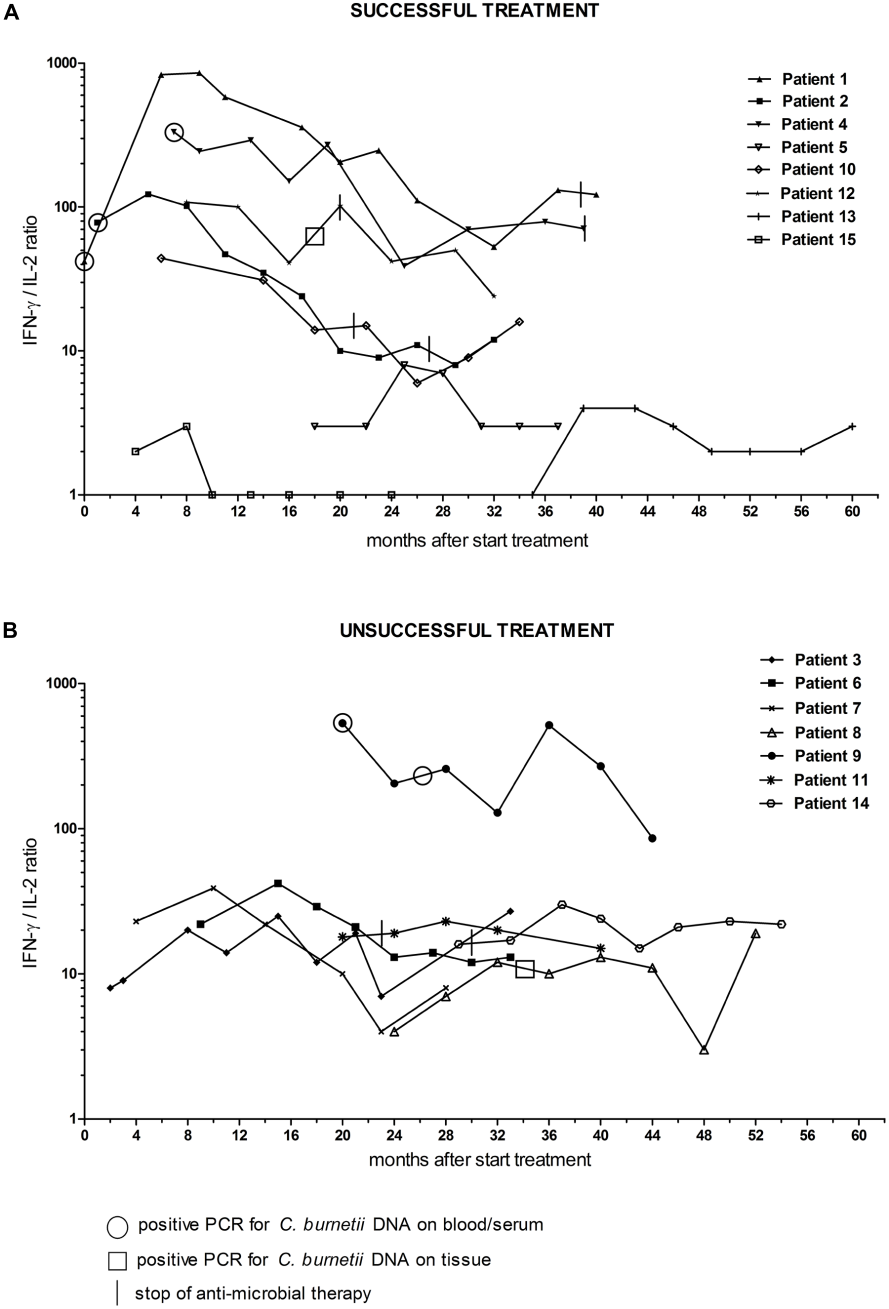
**Interferon-γ/IL-2 ratio in *C. burnetii*-stimulated whole blood of chronic Q fever patients during the study follow-up period, separately shown for **(A)** patients with successful and **(B)** patients with unsuccessful treatment.** *t* = 0 is start of antibiotic treatment. Treatment was considered successful when 18 months of antibiotic treatment (or 24 months when a prosthesis remained *in situ*) was completed, and clinically recovery was observed, and a positive PCR for *C. burnetii* DNA on blood became persistently negative, and anti-phase I IgG showed a fourfold decrease or more (related to the maximum titer), and imaging techniques showed disappearance of any (vascular or valvular) infection focus. Circles indicate a positive PCR on blood/serum, squares indicate positive PCR on tissue. Vertical lines indicate stop of antimicrobial treatment.

**FIGURE 3:**
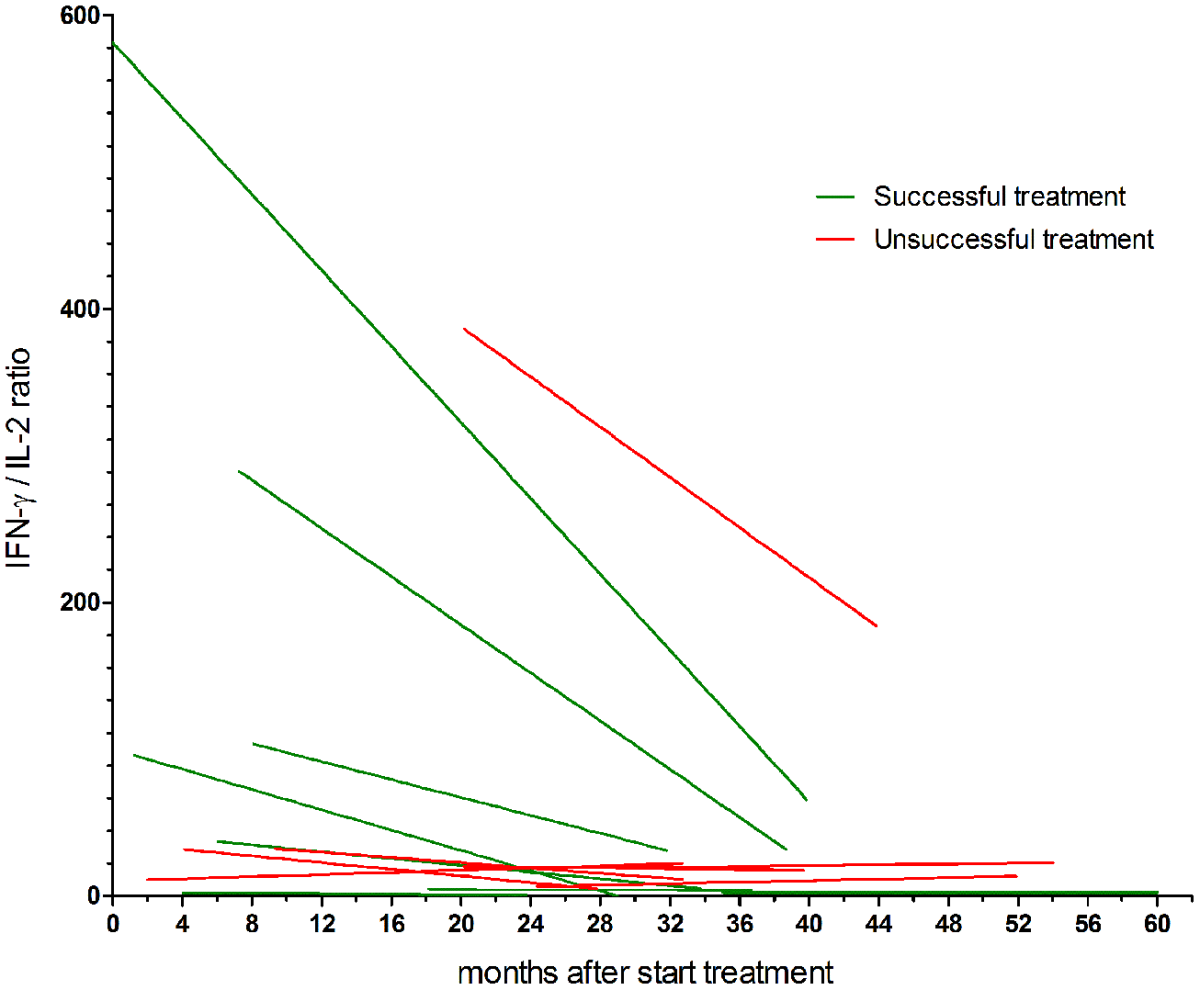
**Non-linear regression to straight curves of the longitudinal IFN-γ/IL-2 ratios of chronic Q fever patients.** Patients with successful treatment are shown in green, patients with unsuccessful treatment are shown in red. The median (±IQR) slope of the patients with successful treatment was -2.10 (-7.02 to -0.06), compared to -0.15 (-1.13 to 0.25) in patients with unsuccessful treatment (*P* = 0.19).

**FIGURE 4:**
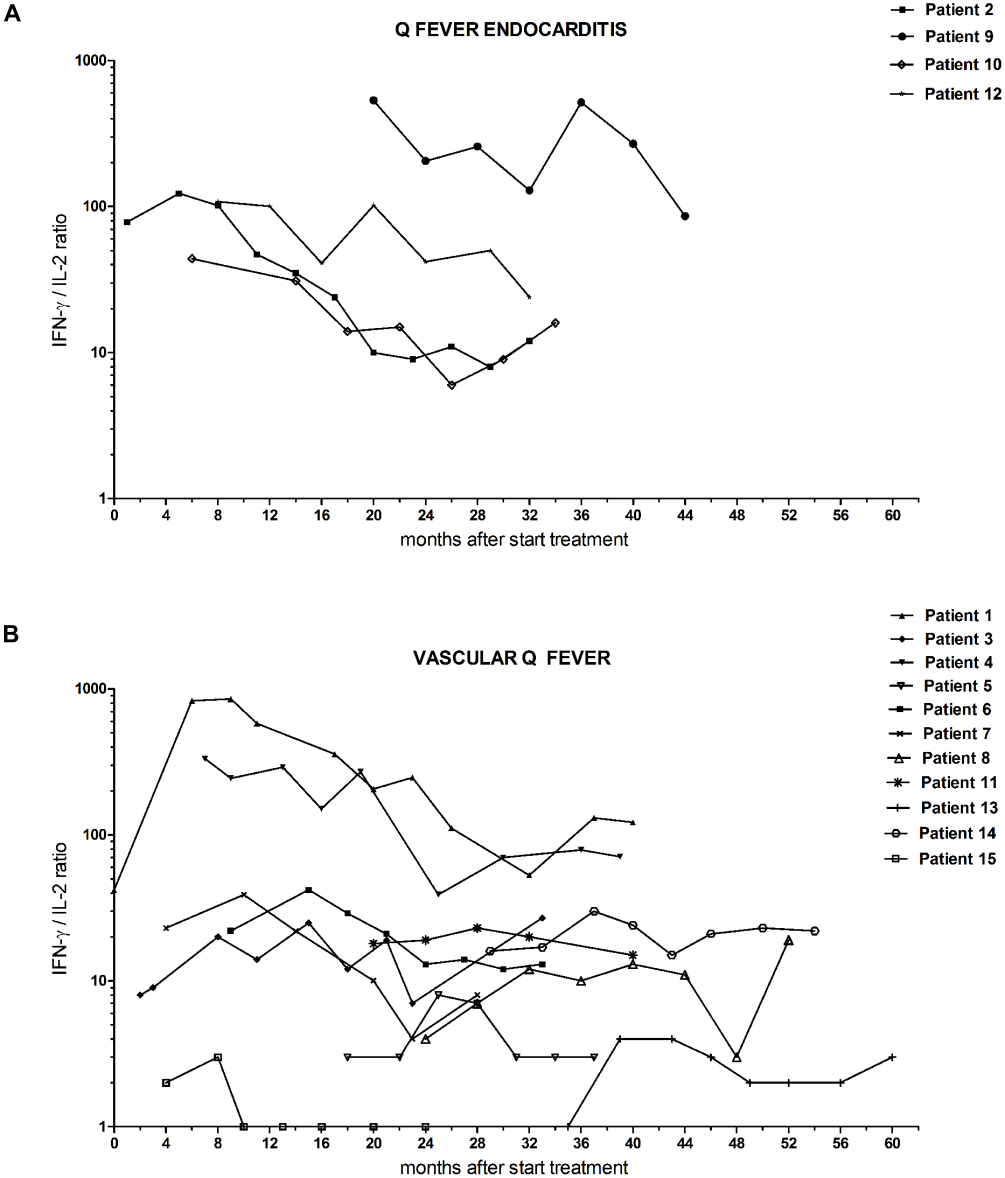
**Follow-up IFN-γ/IL-2 ratio from chronic Q fever patients during the study follow-up period, separately shown for **(A)** patients with endocarditis and **(B)** patients with vascular (prosthesis) infection.** *t* = 0 is start of antibiotic treatment.

We studied the longitudinal IFN-γ/IL-2 ratio in fifteen chronic Q fever patients (**Table 1**). All were followed for at least 18 months during antibiotic treatment. In some cases, this included a period after completion of the treatment. For the purpose of this study, the data of all patients were analyzed according to start of antimicrobial therapy (*t* = 0), which was up to 35 months before inclusion. We divided the group in patients with successful treatment (*n* = 8) and those with unsuccessful treatment (*n* = 7; **Figure 2**). The latter group did not fulfill the success-criteria because of persistent PCR positivity for *C. burnetii* in blood (patient 9), less than a fourfold decrease in anti-phase I IgG titer (patient 3, 8, 11, 14), and/or persistent uptake on FDG-PET/CT (patient 3, 6, 7, 8, 14). As can be seen in **Figure 2**, the patients with successful treatment had higher maximum IFN-γ/IL-2 ratios than those with unsuccessful treatment. Moreover, the IFN-γ/IL-2 ratio of the patients with successful treatment showed a more pronounced decrease, each with an individual pattern, compared to the patients with unsuccessful treatment. We performed non-linear regression of the longitudinal IFN-γ/IL-2 ratios of each patient to a best-fit straight line (**Figure 3**). The median (±IQR) slope of the patients with successful treatment was -2.10 (-7.02 to -0.06), compared to -0.15 (-1.13 to 0.25) in patients with unsuccessful treatment (*P* = 0.19). Because the two main clinical manifestations of chronic Q fever may differ immunologically, the patients with endocarditis were also depicted separately from the patients with vascular (prosthesis) infection (**Figure 4**). Q fever endocarditis patients had overall higher IFN-γ/IL-2 ratios than the vascular Q fever patients.

**Table 1:** Clinical features of the chronic Q fever patients.

Nr	Sex	Age (yrs)^a^	Focus of infection	IgG anti-phase I titre^a,b^	IgG anti-phase II titre^a,b^	PCR serum/ plasma^a^	PCR tissue	Duration of antibiotic treatment (months)^c^
1	Male	30	Vascular graft	65536	65536	Pos	n.a.	0
2	Male	66	Aortic valve	131072	131072	Pos	Pos	1
3	Male	64	Aortic aneurysm	8192	8192	Neg	Pos	2
4	Male	46	Vascular graft	65536	131072	Pos	n.a.	7
5	Female	64	Aortic aneurysm	8192	8192	Pos	n.a.	18
6	Male	72	Vascular graft	16384	8192	Neg	Pos	9
7	Male	74	Aortic aneurysm	65536	32768	Neg	Pos	4
8	Female	54	Vascular graft	4096	4096	Neg	Pos	24
9	Male	79	Prosthetic aortic valve	4096	4096	Pos	n.a.	20
10	Male	58	Mitral valve	4096	8192	Pos	Pos	6
11	Male	67	Vascular graft	4096	4096	Neg	Pos	20
12	Female	65	Prosthetic aortic biovalve	8192	16384	Pos	n.a.	8
13	Male	71	Vascular graft	2048	4096	Pos	Pos	35
14	Male	59	Vascular graft	16384	8192	Pos	Pos	29
15	Male	74	Vascular/Spondylodiscitis	8192	8192	Neg	Pos	4

*n.a., not available; ^a^at the moment of diagnosis of chronic Q fever; ^b^as measured with immunofluorescence assay (IFA, Focus Diagnostics); ^c^at the moment of first blood sample for this study.*

## DISCUSSION

In the present study, we evaluated the usefulness of longitudinal measurements of cell-mediated immune responses against *C. burnetii* for treatment monitoring of chronic Q fever. We measured the *C. burnetii*-specific IFN-γ and IL-2 production in a whole-blood stimulation assay during a period of at least 18 months follow-up of proven chronic Q fever patients. We observed a trend in which the IFN-γ/IL-2 ratio declined when patients experienced a successful outcome of treatment. Patients in whom treatment failed, had overall lower IFN-γ/IL-2 ratios, which did not significantly decrease.

The IFN-γ/IL-2 ratio reflects the type of effector and memory CD4^+^ T-cell response (Sallusto et al., 1999). Memory T lymphocytes can broadly be defined as two distinct populations of effector memory T-cells and central memory T-cells. IFN-γ is predominately produced by effector T-cells and effector memory T-cells, while IL-2 is predominately produced by central memory T-cells. It is postulated that high IFN-γ/IL-2 ratio indicates predominance of effector T-cells and effector memory T-cells, resulting from ongoing immunological stimulation by a persistent infection.

The relevance of measuring IFN-γ/IL-2 production profiles, as diagnostic correlate of memory T-cell responses, has been studied outside the field of Q fever, specifically in a number of viral infections (Younes et al., 2003; Semmo et al., 2005), and in *Mycobacterium tuberculosis* infection (Sargentini et al., 2009; Biselli et al., 2010; Casey et al., 2010; Sester et al., 2011; Essone et al., 2014). These studies, as summarized by Lalvani and Millington (2008), show that in acute and chronic infections with a high antigen concentration, e.g., in chronic progressive HCV infection (Semmo et al., 2005) or untreated tuberculosis (Millington et al., 2007), CD4^+^ T-cells predominately secrete IFN-γ. In infections with persistently low antigen concentrations, e.g., latent asymptomatic cytomegalovirus infection, CD4^+^ T-cells secreting IFN-γ only, IFN-γ/IL-2, or IL-2 only are detected (Harari et al., 2005). In cleared infections, IL-2 secreting CD4^+^ T-cells predominate (Harari et al., 2004, 2005; Correa et al., 2007; Millington et al., 2007). Our finding that the IFN-γ/IL-2 ratio declines during successful treatment of chronic Q fever, assuming a decrease in antigen load, is in accordance with these studies.

The group of patients that was included in our study was inevitably heterogeneous with regard to morbidity, infection status, treatment course and treatment response. Likewise, the inter-individual variation in IFN-γ and IL-2 responses was large. We considered it therefore not feasible to combine individual results for a grouped analysis, and chose to describe patients separately. Three patients were described in more detail, because they were followed (almost) from the start of antibiotic treatment. Strikingly, in two of these patients (patients 1 and 2), who seemed to respond well to treatment, the IFN-γ production and the IFN-γ/IL-2 ratio initially increased, but decreased thereafter. It is tempting to speculate that this initial increase reflects an adequate immune response. A similar initial increase of the specific IFN-γ response is seen in patients during treatment for tuberculosis (Sahiratmadja et al., 2007). The patient that still showed signs of infection after more than 2 years of antibiotic treatment (patient 3), had markedly high IL-2 secretion and a low IFN-γ/IL-2 ratio from the start, which fluctuated in the follow-up but did not decrease. This might suggest bacterial persistence with low antigen concentrations; an assumption that is supported by the notion that *C. burnetii* DNA was not detectable in blood, even before start of antimicrobial therapy. The results of the total group of patients with unsuccessful treatment show the same pattern: overall lower IFN-γ/IL-2 ratio from the start of follow-up in this study, with no *C. burnetii* DNA detectable in blood. An exception to this rule is patient 9, who failed on antibiotic treatment by having *C. burnetii* DNA detectable in blood after 20 months of antimicrobial therapy. The lower IFN-γ/IL-2 ratio we observed in patients with endovascular infections compared to endocarditis patients suggests that these manifestations of chronic Q fever differ with respect to antigen concentrations; vascular infections might be a more low-grade infection than endocarditis.

Our study has some limitations. First of all, we studied a relatively small number of patients. Longer follow-up with additional time points after completion of treatment need to be incorporated into future studies to evaluate success of treatment. Moreover, the method we used, *in vitro* measurement of IFN-γ and IL-2 production, does not clearly differentiate whether these cytokines are produced by effector T-cells (producing only IFN-γ) or effector memory T-cells (producing IFN-γ and IL-2) or central memory T-cells (producing predominantly IL-2). The ratio IFN-γ/IL-2 merely reflects the overall result and might be influenced by the total number of circulating T-cells and their viability *in vitro*. To increase our insight in the matter, detection of cytokine production on single-cell level, e.g., by flow cytometry with intracellular cytokine staining (Harari et al., 2005), would be a valuable addition in future research.

The central position of serology in Q fever diagnostic is undisputable, and serology has hitherto been the most widely used immunological measurement for *C. burnetii* infection. It continues to be extensively validated in Q fever research (Frankel et al., 2011; van der Hoek et al., 2011; Edouard et al., 2013; Herremans et al., 2013). Nevertheless, the immunological importance of measuring antibodies in response to this intracellular bacterial infection is questionable. Specifically, the use of antibody titers to monitor the effect of antibiotic treatment on *C. burnetii* infection and disease needs further research. The definition of serological cure as anti-phase I IgG below 1:800 (or 1:1024 when a commercial IFA is used) is based on expert opinion (Million et al., 2010). The definition of absence of a fourfold decrease in antibody titers as a poor prognostic factor, is based on a small-sized retrospective study of Q fever endocarditis (Million et al., 2010), and is not yet confirmed by other studies. In the light of this limited evidence of serology as a biomarker, it would be valuable to also focus on the more relevant cell-mediated immune response. Especially when the decision to continue or stop treatment has to be made in an individual patient, the availability of other relevant biomarkers may be of help. Our study shows a promising role for the IFN-γ/IL-2 production profile, although the large variation in IFN-γ/IL-2 ratio between patients in this study makes it difficult to formulate general recommendations for the application of these biomarkers at the current time.

In conclusion, existing clinical, imaging and microbiological parameters to monitor the response to treatment have several limitations. We propose that the IFN-γ/IL-2 production profile can be used as an additional immunological biomarker for treatment monitoring of chronic Q fever.

## AUTHOR CONTRIBUTIONS

Conceived and designed the experiments: TS, MGN, JWMvdM, CPBR, MvD. Collected samples: TS, MCWB, MJHP, YEPS, CPBR. Performed the experiments: TS, AA. Analyzed the data: TS, MGN, JWMvdM, CPBR, MvD. TS, JWMvdM, MvD drafted the manuscript and all authors critically revised and approved the final version.

## Conflict of Interest Statement

A patent application has been submitted by Teske Schoffelen, Mihai G. Netea, Marcel van Deuren, Jos W. M. van der Meer, and others to this *Coxiella burnetii* specific IFN-γ and IL-2 production assay to diagnose Q fever and registered by the number PCT/NL 2013/050167. All other authors declare that they have no conflicts of interest.
